# Recent Advances in Implantable 3D-Printed Scaffolds for Repair of Spinal Cord Injury

**DOI:** 10.34172/apb.2024.032

**Published:** 2024-03-10

**Authors:** Salar Khaledian, Ghobad Mohammadi, Mohadese Abdoli, Arad Fatahian, Arya Fatahian, Reza Fatahian

**Affiliations:** ^1^Infectious Diseases Research Center, Health Institute, Kermanshah University of Medical Sciences, Kermanshah, Iran.; ^2^Clinical Research Development Center, Taleghani and Imam Ali Hospitals, Kermanshah University of Medical Sciences, Kermanshah, Iran.; ^3^Pharmaceutical Sciences Research Center, Health Institute, Kermanshah University of Medical Sciences, Kermanshah, Iran.; ^4^Department of Nanobiotechnology, Faculty of Innovative Science and Technology, Razi University, Kermanshah, Iran.; ^5^Nano Drug Delivery Research Center, Health Technology Institute, Kermanshah University of Medical Sciences, Kermanshah, Iran.; ^6^School of Dentistry, Kermanshah University of Medical Sciences, Kermanshah, Iran.; ^7^Department of Neurosurgery, School of Medicine, Kermanshah University of Medical Sciences, Kermanshah, Iran.

**Keywords:** Hydrogel, Polymer-based scaffold, Neural protection, Function recovery, Collagen, Silk

## Abstract

Spinal cord injury (SCI) is an important factor in sensory and motor disorders that affects thousands of people every year. Currently, despite successes in basic science and clinical research, there are few effective methods in the treatment of chronic and acute spinal cord injuries. In the last decade, the use of 3D printed scaffolds in the treatment of SCI had satisfactory and promising results. By providing a microenvironment around the injury site and in combination with growth factors or cells, 3D printed scaffolds help in axon regeneration as well as neural recovery after SCI. Here, we provide an overview of tissue engineering, 3D printing scaffolds, the different polymers used and their characterization methods. This review highlights the recent encouraging applications of 3D printing scaffolds in developing the novel SCI therapy.

## Introduction

**Figure 1:**
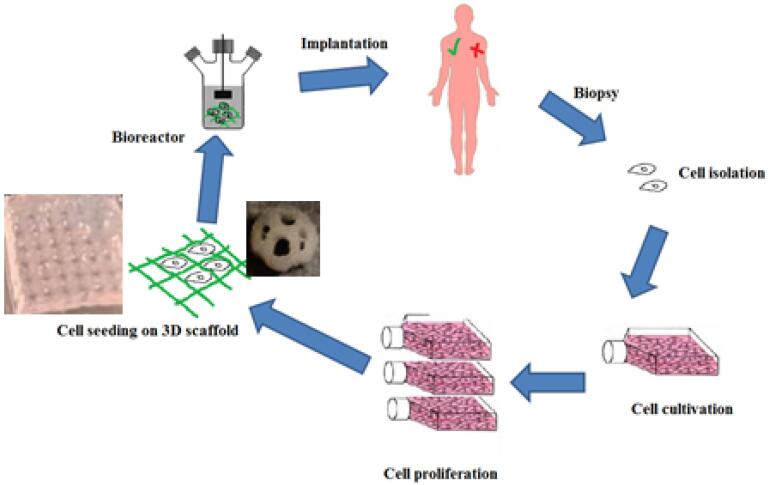
The steps of the tissue engineering process. First, a biopsy of liquid or solid tissue is performed. The cells are isolated using centrifugation or apheresis and then cultured and proliferated. After these cells are implanted in an artificial structure that is similar to the extracellular matrix and can support the formation of 3D tissue. Finally, after passing through the bioreactor stage (simulation a physiological environment), tissue is implanted inside the living body.

 Tissue engineering is a multi-disciplinary science which is used with the aim of creating biological substitutes that can restore, maintain and improve the function of the damaged tissue.^1^ The main components of tissue engineering are scaffold, cells and growth factors.^2^ A tissue has a number of structural and mechanical properties to do its function. For this purpose to obtain these conditions in tissue engineering, the cells are cultured inside an artificial structure. These structures are able to mimic and support the structure of three-dimensional fabric structure. This structure is called a scaffold, which is used both *in vivo* and *ex vivo*. In either case, the scaffold is an imitation of living tissue inside the body, allowing the implanted cells to influence the surrounding microenvironment.^3^ Biological scaffolds are obtained using biocompatible and degradable materials.^4^ As far as possible, the structure of these scaffolds should be as similar to the texture of the planting area. In this way, the reconstruction and improvement of the damaged tissue will increase in quality and quantity. In addition to high mechanical strength, the scaffold structure must have good porosity in order to proper cell nutrition, disposal of cellular lesions to the outside of the scaffold, formation an extracellular matrix, and angiogenesis.^5^ Figure 1 shows a general approach to tissue engineering.

**Figure 2:**
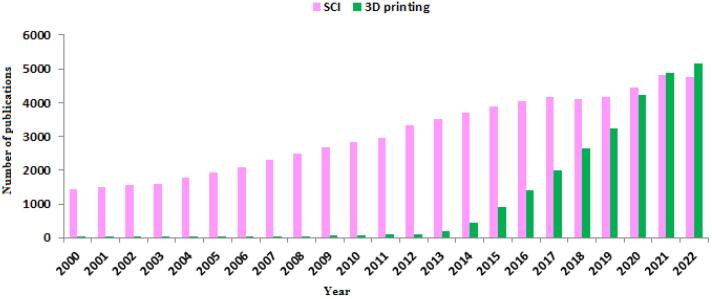
Publication number related to SCI and 3D printing in 2000-2022 years (PubMed* database). In the past decade, the use of 3D printed scaffold in the treatment of SCI has increased significantly.

 Over the past decade, many advances in tissue engineering have been made to repair damaged tissues, including bone, cartilage, skin, cardiac, vascular, liver and nerve tissue.^6^ Spinal cord injuries (SCIs) are relatively common clinical problems that often lead to permanent sensory and motor disabilities in patients. The number of publications about SCI in the last two decades, shows considerable importance of this subject (Figure 2). In the United States, more than 500 000 people suffer from spinal cord injuries, which result in significant psychological and economic costs.^7^ Common treatment strategies for SCI include drug treatment (minocycline, ketorolac, etc), non-drug treatment (decompression, stabilization, etc) and cell transplantation. The prescription of each of these treatment methods depends on the progressive stage of the SCI.^8^ However, scientists and researchers have made extensive efforts to find new and more effective ways to treat this disease. A large number of *in vitro* studies examine cells in a two-dimensional (2D) environment, while in physiological situations in the human body, cells grow in a three-dimensional (3D) environment.^9^ Obviously, in a 2D environment; metabolism, gene expression, role of extracellular matrix (ECM) proteins, and morphology of cultured cells are differed with that occurs under physiological and *in vivo* conditions. As *in vitro* studies become more and more similar to *in vivo* conditions, the construction of three-dimensional cellular networks was developed to replace the damaged tissue.^10^ Combining 3D printing technology with tissue engineering, a new solution was introduced to improve and treat SCI. Scaffolds obtained by classical methods such as particulate leaching, organic foam impregnation, etc., usually have uncontrolled pore size, inadequate high mechanical strength, poor pore geometry and connections. In contrast, scaffolds produced with 3D printers their mechanical properties controlled by pore structure, and their biological activity and degradation are regulated by chemical composition.^11^ Over the past decade, research on 3D printing has accelerated significantly. Therefore, the purpose of this study is to review the applications of 3D printed scaffold in SCI repair.

## 3D printed scaffolds: definitions, types of polymers, synthesis routs, advantages and disadvantages, and so on

 The 3D printing scaffolds are structures with controlled pore size and interconnectivity that are capable of supporting cell growth and tissue formation.^12^ In order to be more efficient, these scaffolds must have critical features such as biocompatibility, biodegradability, mechanical properties and etc.^13^ The first criterion that is of great importance for any tissue engineering scaffold is biocompatibility. Cell binding, migration and proliferation are directly affected by this factor.^14^ Proper biodegradability is another essential feature of scaffolds that in addition to adequate mechanical support prevents the inflammatory response to external scaffold materials. Also, porous structures should facilitate the release of nutrients and metabolic wastes, which in turn increases the metabolic activity of cells. Moreover, the mechanical strength of a scaffold must be sufficient to support the structures in the weight-bearing tissues before the newly formed tissue is synthesized.^15,16^ Eventually, the biomaterials used to prepare tissue engineering scaffolds should be readily available and have the desired fabricability, so that the scaffold produced is compatible with various tissue defects in patients.^13^

**Figure 3:**
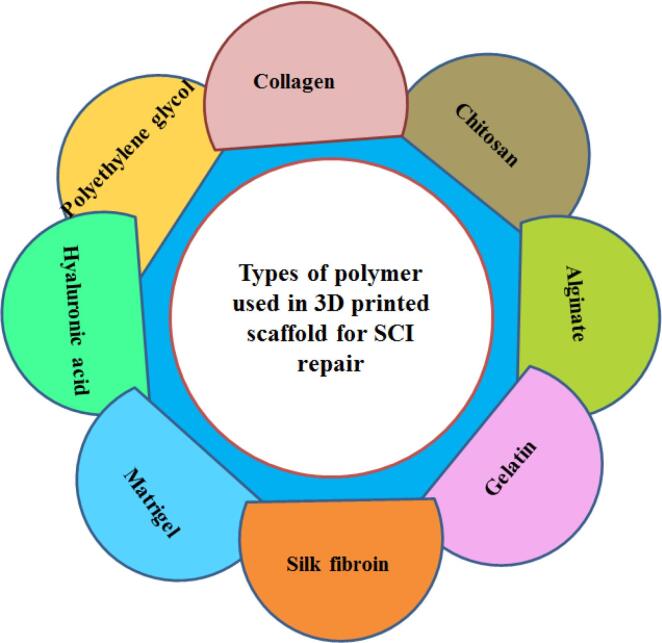
Types of polymer used in the preparation of 3D printed scaffold

 Porosity, pore size, and interconnected pore structure are important factors in 3D printed scaffolds that promote cell survival and enhancing tissue growth.^17^ There are two types of porosity including open (also called interconnected) and closed. In open porosity, the pores are connected like channels, while in closed porosity; the pores are separate and not connected.^18^ Interconnected porosity is very important in tissue engineering and plays an important role in cell nutrition, proliferation, migration and formation of new tissues.^19^ Porosity, in addition to supporting cell migration to the scaffold, improves the available surface for cell attachment to the scaffold and its interaction with the surrounding tissues.^13^ Several studies have shown that scaffolds with sufficient porosity are able to stimulate tissue growth while retaining their mechanical properties.^11,20^ Liu et al fabricated 3D printed scaffolds by collagen and mixture of collagen/chitosan. They compared in this study the microstructures of these scaffolds were compared and a higher total open porosity in the collagen/chitosan case was obtained, which led to improve mechanical strength and facilitate the diffusion of nutrient solution and tissue formation.^20^ In addition, 3D printed scaffolds should have good electrical conductivity to improve the therapeutic effect of SCI. Conductive scaffolds significantly improve the neural differentiation of cells and recovery of motor function.^21^ Finally, the biomaterials must be available and have the desired manufacturing capability to prepare scaffolds with different shapes and suitable for different patients.^13^ Various polymers either naturally derived or synthetic are used for 3D printed scaffolds (Figure 3). In the following, each of these polymers will be discussed briefly.

###  Collagen

 Collagen is one of the main proteins of the ECM, which consists of three alpha polypeptide chains with one or more secondary structures forming a triple helix.^22,23^ The triple helix is usually composed of three polypeptide chains that consist of repeated amino acid units including glycine, proline and 4-hydroxyproline.^24^ Due to hydrogen bonds, the fibrils formed from these triple helixes have high strength and good flexibility and can be connected together, which improves the mechanical properties of 3D printed scaffolds.^25^ The researches on SCI showed that this compound acts as a bridge in the area of the injury and leads to the formation of blood vessels followed by axonal growth.^11^ The results of previous studies in rat models with completely transected spinal cords show that collagen plays a role in reducing lesion area, supporting cell migration, directing orderly regeneration of nerve fibers, and direct axon elongation, all of which improve nerve function.^26,27^ In addition, collagen can act as a delivery carrier for trophic factors and cells to the injury site and form a suitable microenvironment around the injury.^28^ Despite the weak immunogenicity and good biocompatibility of collagen, this polymer faces limitations such as low mechanical properties and high speed of degradation.^29^ One of the strategies presented to increase the mechanical strength and also reduce the rate of degradation is the use of collagen aggregate (extracted from bovine tendon) in the preparation of scaffolds. The results of a study showed that scaffolds made from collagen aggregate increase mechanical strength up to 11 times compared to scaffolds made from collagen molecules.^30^ Chemical modification of collagen through carbodiimide is another way to increase mechanical strength and reduce the rate of degradation by enzymes. Creating bonds between amino groups and free carboxyl groups of collagen fibers improves its mechanical properties.^31^ In this regard, Stiglic et al. reported that the 3D printed scaffold prepared from collagen, carboxymethyl cellulose and nanofibrillated cellulose has high mechanical stability for tissue engineering applications.^32^

###  Chitosan

 Chitosan is a biological polymer that is obtained from chitin as a result of the deacetylation process.^33^ Chitosan, having characteristics such as biodegradability, low toxicity, non-antigenicity, non-hemolysis, and good plasticity, is often used in nerve repair to improve function after SCI.^34,35^ In addition, chitosan has significant neuroprotective properties, maintaining cell membrane integrity and preventing lipid peroxidation during SCI treatment.^36^ Adding chitosan to collagen delays its decomposition and also increases the mechanical properties of the scaffold. However, the degradation time of chitosan is long and it is not suitable for spinal cord transplantation. Hence, a mixture of both substances can overcome the limitations of a pure substance.

###  Alginate

 Alginate is a linear polysaccharide extracted from seaweed that has good stability, solubility, viscosity, and biodegradability.^37^ It is usually used as sodium salt. By adding multivalent cations such as Ca^2+^ and Mg^2+^, which creates ionic bonds between the carboxyl groups of polymer chains, mechanically stable hydrogels were produced.^38^ This key feature can be used especially in tissue engineering and regenerative medicine.^39^ In this regard, various researches have shown that alginate meliorate axon regeneration without causing inflammatory and allergic reactions.^40^

###  Gelatin

 Gelatin with a molecular weight between 15-400 kDa is a very biocompatible and biodegradable protein-based polymer and has many applications in the fields of drug delivery, gene therapy, wound healing, regenerative medicine, and tissue engineering.^41^ This polymer obtained from the collagen through the hydrolysis of natural triple-helix structure of collagen to single-strand molecules.^42^ Compared to collagen, gelatin is less immunogenic and have an important role in the promoting cell adhesion, migration, differentiation and proliferation due to RGD motif.^43^ Furthermore, the combination of gelatin with alginate in 3D printed scaffold improve the mechanical properties and reduce the rate of degradation.^44^

###  Silk fibroin

 Silk fibroin (SF) with molecular weight (200–350 kDa or more) is another natural protein that is produced by different kind of insects and spiders.^45,46^ The most important advantage of SF compared to other natural biopolymers is having excellent mechanical properties. Other advantages of SF include excellent biocompatibility, water-based processing, biodegradability, and the presence of chemical groups for functional changes.^47^ This polymer was used in the soft tissue engineering and reconstruction as a biomaterial.^47,48^ Combination of SF with collagen for preparation of 3D printed scaffold can compensate some limitation of collagen, such as poor mechanical strength, low manipulability and fast degradation.^49,50^

###  Matrigel

 Matrigel^TM^ is a mixture of basement membrane matrix that have been extracted from Englebreth-Holm-Swarm (EHS) tumors in mice.^51^ This protein mixture mostly consisting of laminin, collagen type IV, enactin and various growth factors.^52,53^ Scaffolds prepared from the matrigel are mainly used for cell differentiation, tissue vasculature and angiogenesis.^54,55^ Due to mimicking the ECM components, matrigel could promote cell adhesion on the scaffold.^56,57^ however it has a poor mechanical properties and for application in 3D printing should be combined with another polymers such as alginate, chitosan and etc.^58^

###  Hyaluronic acid

 In two last decades, hyaluronic acid (HA) as a long and natural linear polysaccharide has attracted a lot of attention in the biomedical fields specifically in wound healing and tissue engineering.^59,60^ HA is a mixture of two disaccharides including D-glucuronic and *N*-acetyl-D-glucosamine and have a critical role in different biological processes like a tissue hydration, nutrient diffusion, proteoglycan organization and cell differentiation.^61^ The molecular weight and concentration of HA ranging between 120-2500 kDa and 0.1-4% w/v has been reported respectively for 3D printing application.^62^

###  Polyethylene glycol

 Polyethylene glycol (PEG), a synthetic hydrophilic polymer with biocompatible and non-immunogenic properties that has been widely used in tissue engineering and to improve the wettability of scaffolds.^63,64^ It has been reported that PEG can reseal axonal membranes following SCI. Furthermore, PEG may directly lead to a significant reduction of mitochondrial-derived oxidative stress and prevent its effects on intracellular components.^65^ However, the use of PEG in the fabrication of 3D printing scaffolds has some limitations. The obtained scaffolds do not provide a suitable biological environment for cell proliferation. In addition, cell survival depends on photocrosslinking time, light intensity and photoinitiator.^66^

###  Types of 3D bioprinting technology in SCI repair

 According to the previous research, various 3D printing techniques have been used for treatment of SCI, each of them will be briefly discussed below. 3D bioprinters are classified into 4 categories including extrusion-based, inkjet-based, laser-assisted, and stereolithography-based bioprinting,^67-69^ although other classifications have been reported.^70^

 Among the mentioned systems, extrusion based system is mostly used. In this method pneumatic or mechanical (piston or screw) dispensing systems is used to extrude a continuous bioink stream which leads to excellent structural integrity of the scaffold.^71,72^ This technic can be divided to two category including syringe-based microextrusion or microfluidic extrusion.^73^ In the mentioned system, at first the cells and bio-ink are mixed and then the biomaterials are dispensed through nozzles or needles.^71^ The major advantage of this process are easy application, ability to print different kinds of materials and fabrication of cell density with high values.^74,75^ However, low printing resolution,^76^ low viscosity bioinks^77^ and optimization of parameters like as component concentration, pressure and diameter of the nozzle are among the limitations of this method.^78^

 Inkjet based bioprinting is an affordable method that using a diversity of energy sources produce bioink droplets.^74^ Therefore, there are two types of inkjet printers including thermal and piezoelectric. In these methods by apply the temperature and piezoelectric actuator, air-piezoelectric-pressure pulses were produced and lead to ejection of droplets from the nozzle.^69^ Among the advantages of these methods can be mentioned high printing speed, high resolution (between 20 and 100 µm), and good biocompatibility.^76,79^ But, the blocking of nozzles and the need to use low-viscosity bioinks to solve this problem are among the limitations of these methods, which, by imposing thermal or mechanical pressure, ultimately lead to a decrease in cell viability.^80,81^

 Laser-assisted bioprinting is another promising technology for fabrication of 3D scaffold.^82^ In this method, a variety of bioinks with different viscosity have used without any effects on cell viability and function.^83,84^ In this system, with pressures generated by laser beam, bioink droplets were eject onto a collector substrate which contains culture media to support cell growth.^75^ High printing resolution (≈ 10 µm) and high cell density (~108 cells/mL) are among the advantages of this method.^85^ On the other hand, contaminations with metallic residue reported as a disadvantage of the mentioned system.^86^

 In the stereolithography-based bioprinting, ultraviolet (UV) laser are used for printing of light sensitive substances that polymerize to soft substrate materials.^87^ This method has a high printing resolution (5-50 µm) which depends on various factors including laser power, exposure time, size of the laser spot, and value of light wavelength.^88,89^ However, the mentioned system has a limitations such as slow printing time and restriction in type of bioinks for 3D printing applications.^90,91^

## 3D printed scaffold characterizations: porosity, stability, biodegradability, compatibility (hemo/cytocompatibility) and so on

 Characterization of 3D printed scaffolds are performed by various analysis including scanning electron microscopy−energy dispersive X-ray spectroscopy (SEM−EDS), rheometry, powder X-ray diffraction (PXRD), Fourier-transform infrared spectroscopy (FT-IR), compression tests and etc. After sample drying and sputter-coating with gold, the morphology of 3D printed scaffold is observed by SEM or FE-SEM. Rheometer is used for measure the rheological properties of hydrogels. Two analyzes are used to investigate the mechanical properties of 3D printed scaffolds and compare them with native spinal cord tissue. Atomic force microscopy analysis is used to examine the local mechanics of gray and white matter. Mechanical tester is also used to measure the elastic modulus of 3D printed scaffolds. Finally, the prepared values for native spinal cord tissue and 3D printed scaffolds are compared.^92^ In addition, FTIR spectroscopy is used to confirm the presence (or absence) of functional groups in the scaffold and XRD analysis gives complete structural information about that. Furthermore, the differential scanning calorimetry (DSC) are performed for measuring the thermal stability of fabricated scaffold. For swelling ratio measurement, after immersion the amount of scaffold in phosphate buffered saline (PBS), the weight of the swollen sample is measured.^93^ The swelling ratio (SR) is calculated through a formula below:


(1) $$SR=\frac{W_{s}-W_{d}}{W_{d}}$$

Which* W*_s_ = weights of the swollen sample and *W*_d_ = dry weight of the sample.

 In order to measuring the water absorption ratio, the dried scaffold is swelled in PBS for 1-7 days and after removal free water of the surface, the weight of the sample is measured.^11^ The amount of water absorption (WA) is calculated according to the below equation:


(2) $$WA=\frac{M_{t}-M_{0}}{M_{0}}.100\%$$

WhichM_t _= weights of the swollen sample and M_0 _= dry weight of the sample.

 For porosity ratio, the scaffold is immersed into absolute ethyl alcohol.^11^ Porosity ratio (P) is calculated by following formula:


(3) $$P=\frac{V_{0}-V_{2}}{V_{1}-V_{2}}.100\%$$

Which* V*_0_ = volume of alcohol in the beginning, *V*_1_ = volume of alcohol after negative pressure degassing and *V*_2_ = volume of the residual alcohol after taken out the scaffold.

 For investigation of *in vitro* degradability, the scaffold is incubated in PBS that containing various enzyme such as collagenase, gelatinase, hyaluronidase and among other. Then changes in the wet weight of the scaffold over time is measured.^94^ For *in vivo* degradability, after a longitudinal incision in the dorsal area of the rat, the 3D printed scaffold is implanted into the cystic space.^50^ After 7, 14, 21 and 28 days, the scaffold is removed and degradation rate (D) is calculated using below equation:


(4) $$D=\frac{W_{0}-W_{T}}{W_{0}}.100\%$$

Which* W*_0_ = quality of materials in the beginning and *W*_T _= quality of the residual materials after degrading.

 Finally, cell compatibility of 3D printed scaffold is evaluated by MTT assay. Also, after 3D bioprinting process, cell viability and cell proliferation are assessed by LIVE/DEAD Viability/Cytotoxicity Kit and MTT method.

## 3D printed scaffold applications in spinal cord injury repair

**Figure 4:**
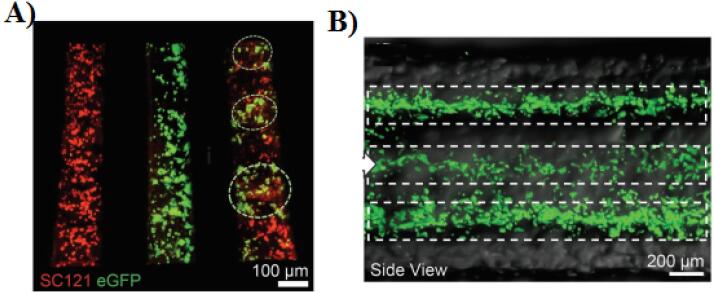
A) Distribution of cell types in specific channels: sNPCs only (left), OPCs only (middle), and sNPCs and OPCs (right). B) longitudinal side view of fluorescence images of a 3D printed scaffold.^95^ Reproduced with permission from John Wiley and Sons.

 Various 3D printed scaffold for SCI repair has been reported in the recent years. In this regards, Li et al designed an in vitro 3D printed sodium alginate-matrigel scaffold to promote the differentiation and growth of ectomesenchymal stem cells. Based on the in vitro results, in addition to better neural differentiation efficiency compared to 2D culture, the cell survival rate on the 3D printed scaffold was more than 88%.^58^ In another study, Joung et al produced 3D printed scaffold based on matrigel/gelatin/GelMa with microextrusion-based method. Their results showed that the sNPCs and OPCs were distributed in specific channels (Figure 4A) and cells were alive for 3 days after printing in all three layers (Figure 4B) which indicates that this 3D printed scaffolds are neurocompatible for both cell types.^95^

**Figure 5:**
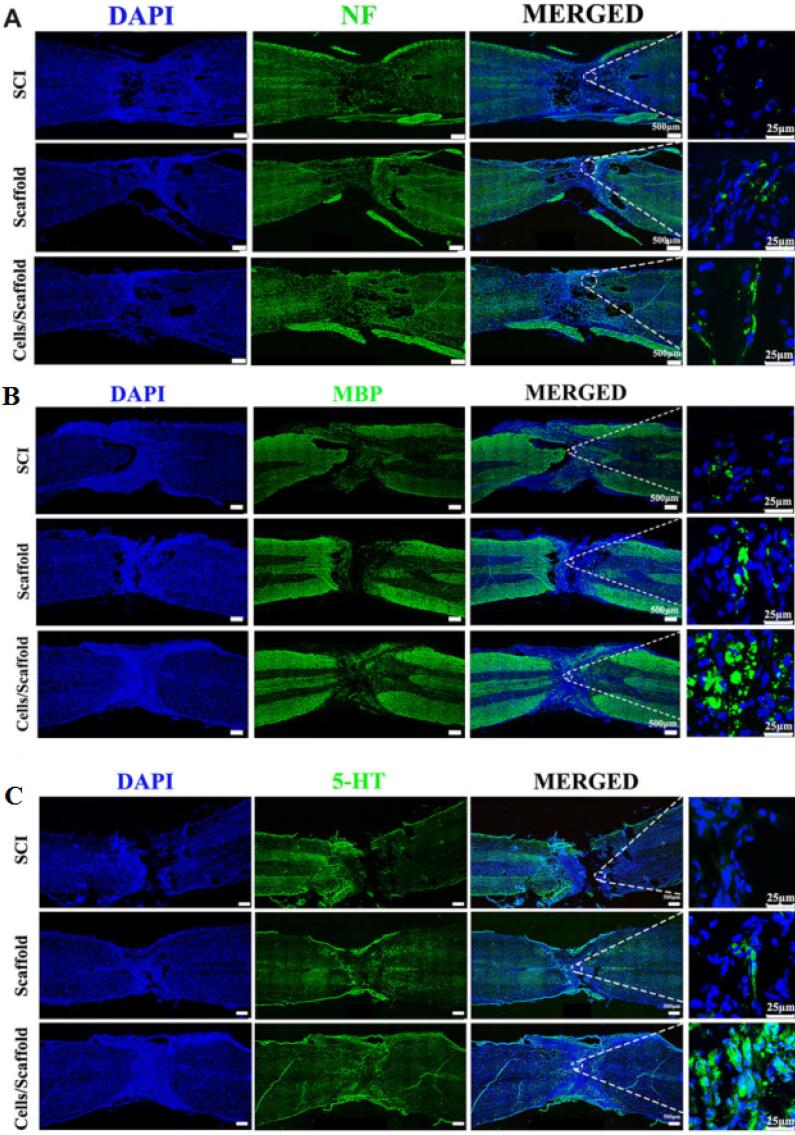
3D bioprinting scaffold with NSCs and OLGs promoted myelinization and regeneration of axons. Immunofluorescence staining against NF (A), MBP (B) and 5-HT (C). Reproduced from Liu et al^44^ under the terms of the Creative Commons Attribution License (CC BY 4.0)

 Lio and colleagues fabricated a 3D printed sodium alginate/gelatin scaffold combined with neural stem cells (NSCs) and oligodendrocytes (OLGs) and investigated their effects on nerve regeneration after SCI.^44^ Based on their results, after 3 and 5 days the mean cell survival was about 83% and 76%. The prepared scaffolds (volume about 3 × 2 × 3 mm^3^ containing 2 × 10^4^ cell) implanted into the transected rat spinal cord for 8 weeks. The *in vivo* results showed that the 3D printed scaffold loaded with both cells compared to other groups (SCI and Scaffold) obviously ameliorated neural generation, axon growth, and locomotor functional recovery (Figure 5).

**Figure 6:**
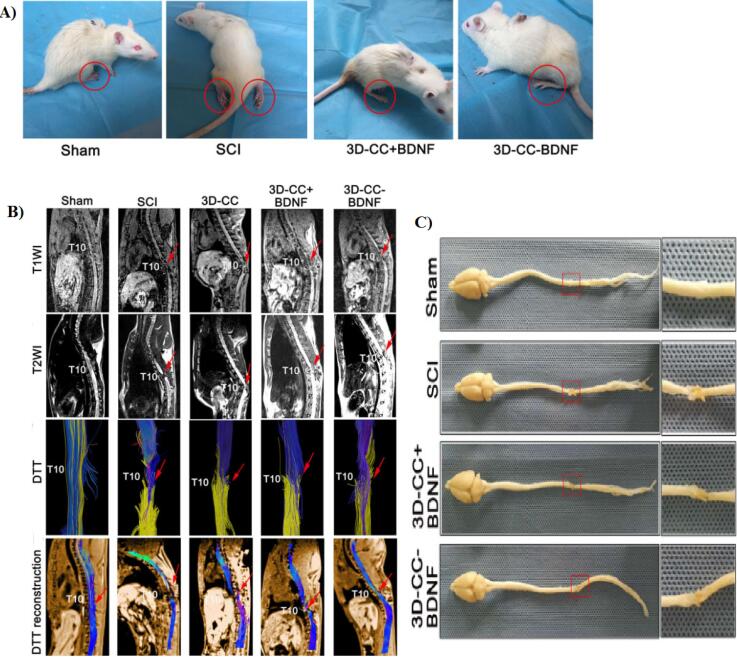
A) Typical picture of rats in the four groups at 8 week after the operation. B) Magnetic resonance imaging (MRI) and diffusion tensor imaging (DTI) at 8 weeks after SCI. C) Macroscopic histology images of spinal cord. Reproduced from Liu et al^20^ under the terms of the Creative Commons Attribution Non-Commercial License (https://creativecommons.org/licenses/by-nc/4.0/)

 Liu et al^96^ produced NSC-laden scaffolds using microextrusion-based 3D printing system. They announced that the cell survival rate was more than 95%. In addition, their result showed that 3D printed scaffold (volume about 4 × 2 × 2 mm^3^ containing 3 × 10^5^ cell) providing a good microenvironment for cell growth and neural differentiation. Furthermore, after implantation for 12 weeks, the locomotor function in rat model was improved by promoting the axon regeneration and reduced glial scar deposition. In another study, Lio and co-worker constructed a BDNF/collagen/chitosan scaffold using an extrusion 3D printer.^20^ Their results showed that combination of collagen and chitosan leads to improvement of physicochemical properties of scaffold including mechanical strength, elastic modulus, porosity ratio and etc. In addition, NSCs or HUCMSCs grew fine on this scaffold, which indicating a good cytocompatibility of scaffold. Based on the in vivo experiments, implantation of 3D-CC-BDNF for 8 weeks ameliorated locomotor function after SCI (Figure 6A). Furthermore, by transplantation of the mentioned scaffold after SCI the regeneration of nerve fibers tracts were promoted (Figure 6B). Also, with the implantation of prepared scaffold a) the gap at the site of injury is filled, b) the regeneration of the nerve fiber is facilitated and c) the creation of synaptic connections is accelerated.

**Figure 7:**
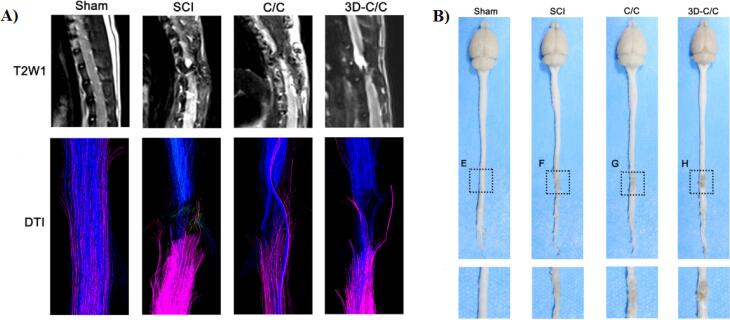
A) MRI-DTI evaluating the recovery of white matter fiber after SCI. B) The spinal cord and brain were retrieved after perfusion of 4% formaldehyde at 8 weeks post-surgery.^11^ Reproduced with permission from John Wiley and Sons.

 Sun and colleagues in 2019 produced a 3D printed collagen/chitosan (3D-C/C) scaffold for treatment of SCI.^11^ In this study, 3D-C/C scaffold is implanted into the T8 complete transection SCI rat model. Their results showed that compared to other group (SCI or C/C group), 3D-C/C scaffold implantation leads to improving the locomotor function in SCI rat. Based on MRI-DTI images, using this scaffold and compared with SCI group the spinal cord fibers around the lesion site were significant elevated (Figure 7A). Also, they stated that after 8 weeks implantation of 3D-C/C scaffold the scar and cavity production were decreased and the regeneration of nerve fibers were improved (Figure 7B).

**Figure 8:**
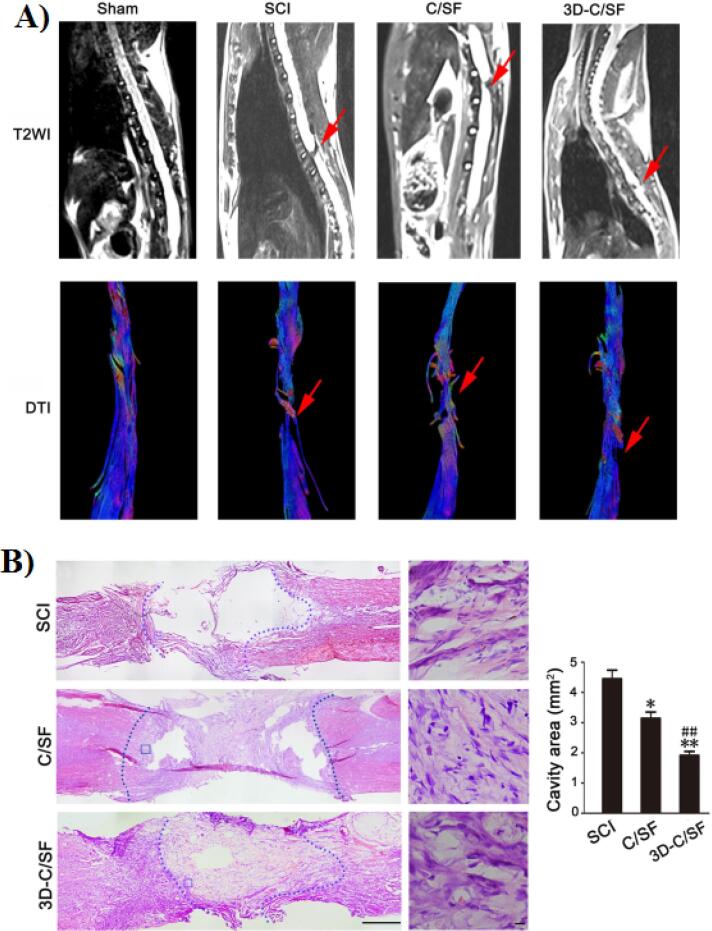
A) MRI images of spinal cord in each group at 8 weeks after implantation. B) HE staining at 8 weeks post injury. Reproduced from Li et al^50^ under the terms of the Creative Commons Attribution License (CC BY 4.0)

 Li et al developed a 3D printed collagen/silk fibroin (3D-C/SF) scaffold for the treatment of SCI.^50^ In this study, the completely transected SCI rats were implanted with 3D-C/SF scaffold and their functional recovery was evaluated after 8 weeks. Their results showed that this 3D printed scaffold promoted the locomotor function after SCI. Also, based on the MRI results in the 3D-C/SF group compared to SCI and C/SF group the axonal regeneration was significantly improved (Figure 8A). In addition, histological analysis showed that lesions and disordered structures were fewer in 3D-C/SF scaffold compared to other groups (Figure 8B).

**Figure 9:**
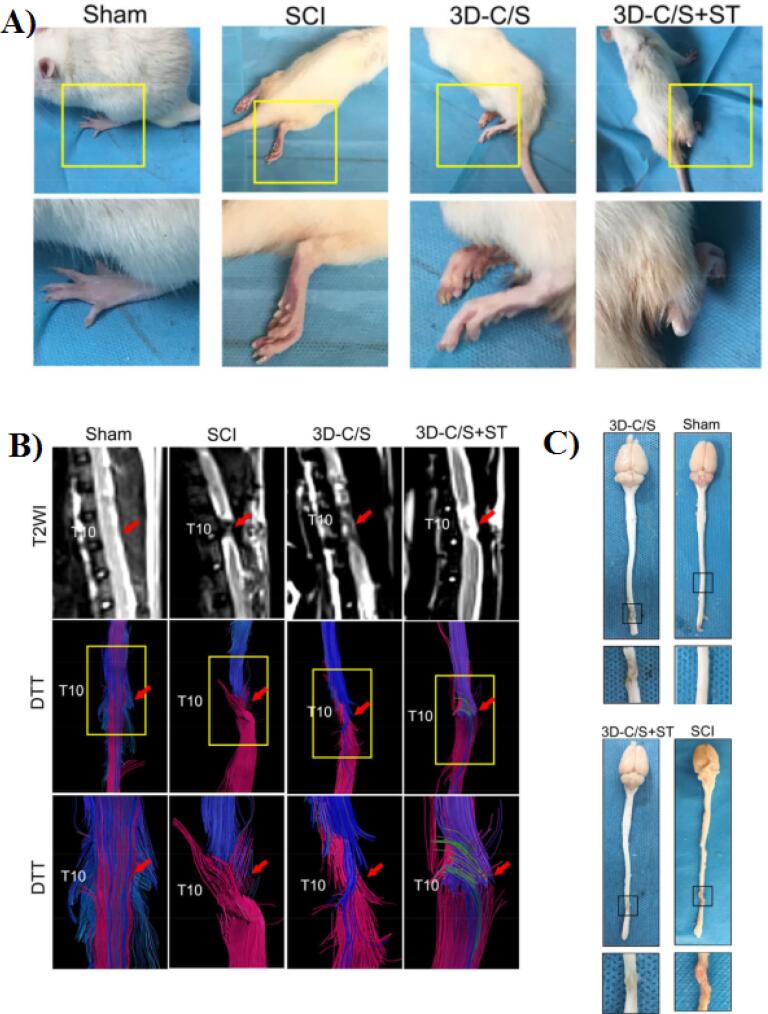
A) Representative gait records of walking gaits of hindlimbs of rats in each group at 8 weeks after surgery. B) MRI and DTI images at 8 weeks after surgery. C) Representative macrograph of spinal cord at 8 weeks post-surgery. Reproduced from Chen et al^49^ under the terms of the Creative Commons Attribution License (CC BY 4.0)

 In another work, Jiang and co-workers fabricated a 3D printed collagen/silk fibroin scaffold loaded with NSCs for nerve regeneration after SCI.^97^ Based on the results, the NSCs exhibited strong proliferation and differentiation ability. In addition, in the 3D-CF + NSCs groups the locomotor function of SCI rats were improved at 8 weeks after surgery. Also, the cavity in the injury site was filled with 3D printed scaffold and regeneration of nerve fibers enhanced. Chen and colleagues in 2022 developed a 3D printed collagen/silk fibroin scaffolds combined with secretome of human umbilical mesenchymal stem cells (3D-C/S + ST).^49^ In comparison to 3D-C/S scaffold, the 3D-C/S + ST scaffold showed a good cytocompatibility and was better for NSCs growth. Based on their results 8 weeks after surgery, the locomotor function as well as the hindlimb walking ability of 3D-C/S + ST group were notably improved (Figure 9A). In addition, MRI and DTI image indicates that after implantation of 3D-C/S + ST scaffold into the T10 complete transection SCI rat model, the nerve fibers tracts regeneration was enhanced (Figure 9B). Also, the implanted scaffold significantly decreased cavity formation, improve nerve fiber regeneration and remyelination at the injury site (Figure 9C).

 Koffler et al in 2019 fabricated a 3D PEG–GelMA printed scaffolds loaded with neural progenitor cells (NPCs) for SCI repair.^7^ In this study the prepared scaffold implanted into SCI rats. Based on their results, after 6 months, in every grafted animal the NPC cells survived and completely filled the channels of the scaffold. Furthermore, in vivo results indicates that 3D printed scaffolds combined with NPCs significantly enhance host axonal regeneration into the lesion. Based on the anatomical analysis, the scaffolds maintained their 3D architecture after 6 months. In addition, the functional recovery of animal that received scaffolds loaded with NPCs were improved compared to animals with empty scaffolds.

 In another study, the T3 complete transection SCI rat models were implanted with 3D PEG-GelMA scaffold loaded with NPCs and compared with 3D-printed scaffolding made of HA, traditional agarose scaffolding, or NPCs without a scaffold. Their results showed that after 4 weeks all NPCs survived and axon regeneration and myelination improved.^98^

 Yang *et al* produced a 3D printing scaffold containing NSCs for long-distance SCI regeneration.^94^ Their results showed that the viability of cells after 7 day of printing were more than 94%. In addition, the implantation of scaffold in SCI rat model significantly enhanced nerve and myelin regeneration. Also, this system reconstructs a functional network at the injury site, which was the essential situation for functional recovery.

**Figure 10:**
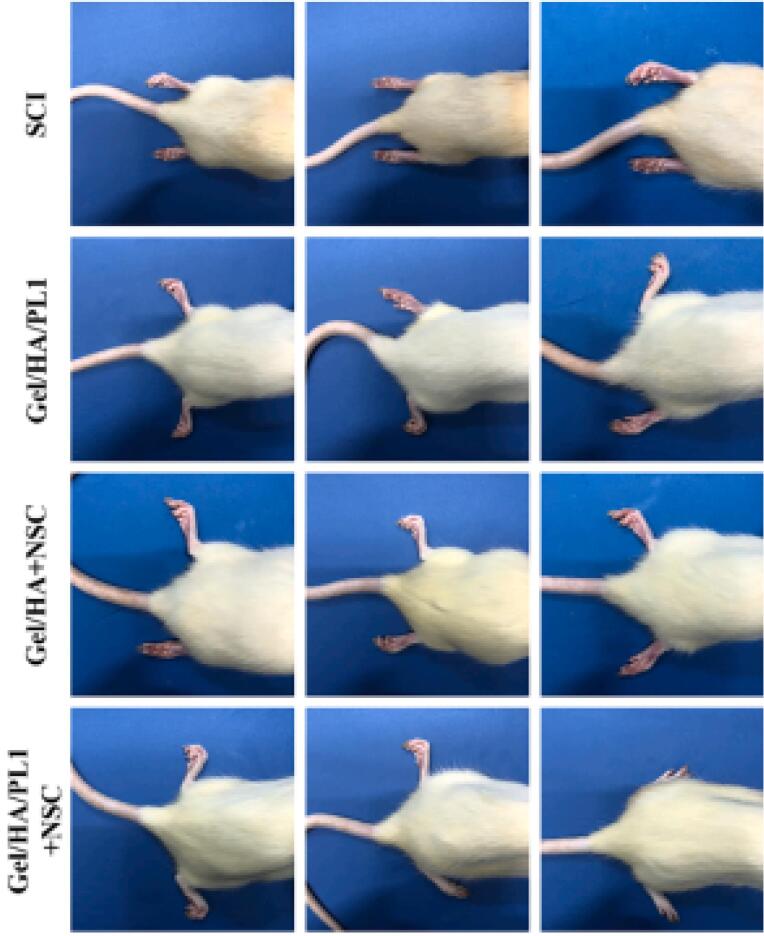
Typical hindlimbs-spreading status of SCI rats on week 8 post-surgery^21^ Reproduced with permission from Elsevier.

 Gao and co-worker developed a 3D printed conductive hydrogels based on GelMA, HAMA, and PEDOT:LS.^21^ Based on their results, the NSCs viability was more than 90%. *In vitro* results show that the prepared scaffolds significantly promoted neuronal differentiation of NSCs. Also, implantation of prepared 3D printed scaffold leads to improving the locomotor function in SCI rat model (Figure 10). Furthermore, the use of these 3D printed scaffolds reduces glial scar deposition, and promotes the regeneration of nerve axons and myelination in the injury site.

## Concluding and future perspective

 Currently, the treatment of SCI is considered as an important and fundamental challenge in the world. The obvious reason for the lack of treatment is the poor recovery ability of the central nervous system. Various chemical and physical factors are effective in preventing the reconstruction of SCI. Inhibitory chemical barriers at the site of SCI include inflammatory factors, free radicals of oxygen, inhibitory amino acids and neurotransmitters, which easily lead to toxic reactions and exacerbation of local edema, resulting in insufficient blood supply and accumulation of neurotrophic substances. Furthermore, the loss of spinal cord tissue and the formation of hypertrophic glial scar are physical obstacles to the regeneration of axons. Many evidences show that the implant of biomaterials is a successful strategy for neurological treatment after SCI. Scaffolds that are prepared by traditional methods such as particulate leaching, organic foam impregnation, pore-forming agent, freeze-drying, etc. have an amorphous and irregular structure and are not suitable for axon regeneration at the lesion site. Scaffolds prepared with 3D printing technology have a several advantages including:

1. Good biodegradability (more than 50% of the scaffolds are decomposed after 8 weeks of implantation) without significant inflammatory reaction
2. Having a typical shear-thinning behavior to protect cells from shear force damage during the printing process
3. Excellent cell compatibility and cell adhesion ability
4. Having a porous structure that can increase oxygen, nutrition, and waste transfer
5. Improve the cell-matter and cell-cell interactions, tissue integration and rapid vascularization
6. High potential in regenerating axons by improving the chemical microenvironment and physical barriers
7. Help to reduce the scars and cavity formation
8. Improve the regeneration of nerve fibers and also functional recovery

Scaffold implants (between 3-10 vertebrae) are performed either immediately after the creation of the lesion or after a period of time has passed since the creation of the lesion; in the second case, due to the production of glial scars, the repair of the SCI faces a problem. Different synthetic and natural polymers are used to prepare 3D scaffolds. Scaffolds prepared with natural polymers compared to scaffolds prepared with synthetic polymers (I) improve the adhesion ability of implanted cells, (II) have good biocompatibility and degradation, (III) non-toxic, (IV) increase the survival time of implanted cells inside the body, (V) increase the rate of differentiation of implanted cells into nerve cells, and (VI) improve tissue regeneration and functional rehabilitation.

 The use of loaded growth factors in scaffolds is another solution for SCI treatment. The implant of these scaffolds in the lesion site and the gradual release of growth factors are significantly effective in neuron regeneration and subsequent functional recovery. In addition, growth factors help to decrease cavity and glial scar formation. 3D scaffolds containing cells compared to scaffolds without cells and scaffolds containing growth factors notably improve neuron regeneration and motor functional recovery. The degree of stiffness of the scaffolds, which can be adjusted by changing the concentration of the components in bioink, is effective in the differentiation of NSCs into neurons or astrocytes. In this case, if the amount of elastic modulus is less than 1 kPa, the NSCs cells become neurons, and if it is 3 kPa or more, an obvious reduction of differentiated neurons occurs. Cell grafts can rarely viable for a long time because of the presence of inflammatory and toxic blood products at the injury site, however the cells loaded in the 3D scaffolds survive at the site of the lesion for more than 12 weeks and differentiated to neurons and oligodendrocytes, while cells without scaffolds barely survived for 4 weeks. The NSCs cells loaded in the scaffold can differentiate into functional neurons related to movement and sensation, which ultimately improves functional recovery. According to research results, axon regeneration alone is not enough to restore motor functions, and in the regeneration process, another key factor is remyelination of regenerated axons. In this regards, the simultaneous use of two types of cells, NSCs and OLGs, in combination with 3D printing scaffolds has been beneficial in promoting remyelination and improving the therapeutic effect of SCI. While 3D printed scaffolds containing cells help in neurological regeneration, as well as protected the rat from hard urinary tract complications caused by SCI. In addition, the prepared scaffolds are safe for other organs like as lungs, liver, spleen and heart. According to the results of various studies to improve the efficiency of 3D printed scaffolds containing cells in SCI repair as well as their use in the clinical phase in the near future, the following are suggested.

- In clinical application, using polymers with complex structure and variable components such as matrigel has a limitation and for this reason it is better replacing it with other polymers such as laminin and collagen type 4.
- Simultaneous use of two or more cells and growth factors in order to better and faster reconstruction of SCI.

## Competing Interests

 The authors declare that they have no known competing financial interests or personal relationships that could have appeared to influence the work reported in this paper.

## Ethical Approval

 Not applicable.
